# Supplementary material for: OpenMM 7: Rapid development of high performance algorithms for molecular dynamics
Source: PLoS Comput Biol. 2017 Jul 26;13(7):e1005659. doi: 10.1371/journal.pcbi.1005659 (PMC5549999; doi:10.1371/journal.pcbi.1005659)
Supplement: S1 Source Code — (ZIP) [file pcbi.1005659.s002.zip › OpenMM-7.0.1-Source/docs/api-python/generated/simtk.openmm.openmm.Platform.html]

Platform — OpenMM 7.0.1.dev-5e86c4f documentation


# Platform¶

*class* `simtk.openmm.openmm.``Platform`(*\*args*, *\*\*kwargs*)¶
:   A Platform defines an implementation of all the kernels needed to perform some calculation. More precisely, a Platform object acts as a registry for a set of KernelFactory objects which together implement the kernels. The Platform class, in turn, provides a static registry of all available Platform objects.

    To get a Platform object, call

    <pre>
    Platform& platform = Platform::findPlatform(kernelNames);
    </pre>

    passing in the names of all kernels that will be required for the calculation you plan to perform. It will return the fastest available Platform which provides implementations of all the specified kernels. You can then call createKernel() to construct particular kernels as needed.

    `__init__`(*\*args*, *\*\*kwargs*)¶

    Methods

    |  |  |
    | --- | --- |
    | `__init__`(\*args, \*\*kwargs) |  |
    | `findPlatform`((kernelNames) -> Platform) | Find a Platform which can be used to perform a calculation. |
    | `getDefaultPluginsDirectory`(...) | Get the default directory from which to load plugins. |
    | `getName`((self) -> std::string const &) | Get the name of this platform. |
    | `getNumPlatforms`(() -> int) | Get the number of Platforms that have been registered. |
    | `getOpenMMVersion`(() -> std::string const &) | Get a string containing the version number of the OpenMM library. |
    | `getPlatform`((index) -> Platform) | Get a registered Platform by index. |
    | `getPlatformByName`((name) -> Platform) | Get the registered Platform with a particular name. |
    | `getPluginLoadFailures`(() -> vectorstring) | Get any failures caused during the last call to loadPluginsFromDirectory |
    | `getPropertyDefaultValue`((self, ...) | Get the default value of a Platform-specific property. |
    | `getPropertyNames`((self) -> vectorstring) | Get the names of all Platform-specific properties this Platform supports. |
    | `getPropertyValue`((self, context, ...) | Get the value of a Platform-specific property for a Context. |
    | `getSpeed`((self) -> double) | Get an estimate of how fast this Platform class is. |
    | `loadPluginLibrary`(file) | Load a dynamic library (DLL) which contains an OpenMM plugin. |
    | `loadPluginsFromDirectory`(...) | Load multiple dynamic libraries (DLLs) which contain OpenMM plugins from a single directory. |
    | `registerPlatform`(platform) | Register a new Platform. |
    | `setPropertyDefaultValue`(self, property, value) | Set the default value of a Platform-specific property. |
    | `setPropertyValue`(self, context, property, value) | Set the value of a Platform-specific property for a Context. |
    | `supportsDoublePrecision`((self) -> bool) | Get whether this Platform supports double precision arithmetic. |
    | `supportsKernels`((self, kernelNames) -> bool) | Determine whether this Platforms provides implementations of a set of kernels. |

    *static* `registerPlatform`(*platform*)¶
    :   Register a new Platform.

    *static* `getNumPlatforms`() → int¶
    :   Get the number of Platforms that have been registered.

    *static* `getPlatform`(*index*) → Platform¶
    :   Get a registered Platform by index.

    *static* `getPluginLoadFailures`() → vectorstring¶
    :   Get any failures caused during the last call to loadPluginsFromDirectory

    *static* `getPlatformByName`(*name*) → Platform¶
    :   Get the registered Platform with a particular name. If no Platform with that name has been registered, this throws an exception.

    *static* `findPlatform`(*kernelNames*) → Platform¶
    :   Find a Platform which can be used to perform a calculation.

        |  |  |
        | --- | --- |
        | Parameters: | **kernelNames** (*vector< std::string >*) – the names of all kernels which will be needed for the calculation |
        | Returns: | the fastest registered Platform which supports all of the requested kernels. If no Platform exists which supports all of them, this will throw an exception. |
        | Return type: | Platform |

    *static* `loadPluginLibrary`(*file*)¶
    :   Load a dynamic library (DLL) which contains an OpenMM plugin. Typically, each Platform is distributed as a separate dynamic library. This method can then be called at runtime to load each available library. Each library should contain an initializer function to register any Platforms and KernelFactories that it contains.

        If the file does not exist or cannot be loaded, an exception is thrown.

        |  |  |
        | --- | --- |
        | Parameters: | **file** (*string*) – the path to the dynamic library file. This is interpreted using the operating system’s rules for loading libraries. Typically it may be either an absolute path or relative to a set of standard locations. |

    *static* `loadPluginsFromDirectory`(*directory*) → vectorstring¶
    :   Load multiple dynamic libraries (DLLs) which contain OpenMM plugins from a single directory. This method loops over every file contained in the specified directory and calls loadPluginLibrary() for each one. If an error occurs while trying to load a particular file, that file is simply ignored. You can retrieve a list of all such errors by calling getPluginLoadFailures().

        |  |  |
        | --- | --- |
        | Parameters: | **directory** (*string*) – the path to the directory containing libraries to load |
        | Returns: | **vector< std** – the names of all files which were successfully loaded as libraries |
        | Return type: | :string > |

    *static* `getDefaultPluginsDirectory`() → std::string const &¶
    :   Get the default directory from which to load plugins. If the environment variable OPENMM\_PLUGIN\_DIR is set, this returns its value. Otherwise, it returns a platform specific default location.

        |  |  |
        | --- | --- |
        | Returns: | the path to the default plugin directory |
        | Return type: | string |

    *static* `getOpenMMVersion`() → std::string const &¶
    :   Get a string containing the version number of the OpenMM library.

    `getName`(*self*) → std::string const &¶
    :   Get the name of this platform. This should be a unique identifier which can be used to recognized it.

    `getSpeed`(*self*) → double¶
    :   Get an estimate of how fast this Platform class is. This need not be precise. It only is expected to return an order or magnitude estimate of the relative performance of different Platform classes. An unoptimized reference implementation should return 1.0, and all other Platforms should return a larger value that is an estimate of how many times faster they are than the reference implementation.

    `supportsDoublePrecision`(*self*) → bool¶
    :   Get whether this Platform supports double precision arithmetic. If this returns false, the platform is permitted to represent double precision values internally as single precision.

    `getPropertyNames`(*self*) → vectorstring¶
    :   Get the names of all Platform-specific properties this Platform supports.

    `getPropertyValue`(*self*, *context*, *property*) → std::string const &¶
    :   Get the value of a Platform-specific property for a Context.

        |  |  |
        | --- | --- |
        | Parameters: | - **context** (*Context*) – the Context for which to get the property - **property** (*string*) – the name of the property to get |
        | Returns: | the value of the property |
        | Return type: | string |

    `setPropertyValue`(*self*, *context*, *property*, *value*)¶
    :   Set the value of a Platform-specific property for a Context.

        |  |  |
        | --- | --- |
        | Parameters: | - **context** (*Context*) – the Context for which to set the property - **property** (*string*) – the name of the property to set - **value** (*string*) – the value to set for the property |

    `getPropertyDefaultValue`(*self*, *property*) → std::string const &¶
    :   Get the default value of a Platform-specific property. This is the value that will be used for newly created Contexts.

        |  |  |
        | --- | --- |
        | Parameters: | **property** (*string*) – the name of the property to get |
        | Returns: | the default value of the property |
        | Return type: | string |

    `setPropertyDefaultValue`(*self*, *property*, *value*)¶
    :   Set the default value of a Platform-specific property. This is the value that will be used for newly created Contexts.

        |  |  |
        | --- | --- |
        | Parameters: | - **property** (*string*) – the name of the property to set - **value** (*string*) – the value to set for the property |

    `supportsKernels`(*self*, *kernelNames*) → bool¶
    :   Determine whether this Platforms provides implementations of a set of kernels.

        |  |  |
        | --- | --- |
        | Parameters: | **kernelNames** (*vector< std::string >*) – the names of the kernels of interests |
        | Returns: | true if this Platform provides implementations of all the kernels in the list, false if there are any which it does not support |
        | Return type: | bool |

    `__delattr__`¶
    :   x.\_\_delattr\_\_(‘name’) <==> del x.name

    `__format__`()¶
    :   default object formatter

    `__getattribute__`¶
    :   x.\_\_getattribute\_\_(‘name’) <==> x.name

    `__hash__`¶

    `__reduce__`()¶
    :   helper for pickle

    `__reduce_ex__`()¶
    :   helper for pickle

    `__sizeof__`() → int¶
    :   size of object in memory, in bytes

    `__str__`¶

High performance molecular simulation on GPUs


### Navigation

- Application Layer
- Library Layer
  - Core Objects
  - Forces
  - Integrators
  - Extras

©2015, Stanford University and the Authors.
|
Powered by Sphinx 1.3.1
& Alabaster 0.7.7
|
Page source
